# Association of mitochondrial DNA copy number with prevalent and incident type 2 diabetes in women: A population-based follow-up study

**DOI:** 10.1038/s41598-021-84132-w

**Published:** 2021-02-25

**Authors:** Ashfaque A. Memon, Jan Sundquist, Anna Hedelius, Karolina Palmér, Xiao Wang, Kristina Sundquist

**Affiliations:** Wallenberg Laboratory, Center for Primary Health Care Research, Skåne University Hospital, Lund University/Region Skåne, 6th Floor, Inga Marie Nilsson’s gata 53, 20502 Malmö, Sweden

**Keywords:** Biomarkers, Molecular medicine, Endocrine system and metabolic diseases

## Abstract

Mitochondrial dysfunction is an important factor of the aging process and may play a key role in various diseases. Mitochondrial DNA copy number (mtDNA-CN) is an indirect measure of mitochondrial dysfunction and is associated with type 2 diabetes mellitus (T2DM); however, whether mtDNA-CN can predict the risk of developing T2DM is not well-known. We quantified absolute mtDNA-CN in both prevalent and incident T2DM by well-optimized droplet digital PCR (ddPCR) method in a population-based follow-up study of middle aged (50–59 years) Swedish women (n = 2387). The median follow-up period was 17 years. Compared to those who were free of T2DM, mtDNA-CN was significantly lower in both prevalent T2DM and in women who developed T2DM during the follow-up period. Mitochondrial DNA-copy number was also associated with glucose intolerance, systolic blood pressure, smoking status and education. In multivariable Cox regression analysis, lower baseline mtDNA-CN was prospectively associated with a higher risk of T2DM, independent of age, BMI, education, smoking status and physical activity. Moreover, interaction term analysis showed that smoking increased the effect of low mtDNA-CN at baseline on the risk of incident T2DM. Mitochondrial DNA-copy number may be a risk factor of T2DM in women. The clinical usefulness of mtDNA-CN to predict the future risk of T2DM warrants further investigation.

## Introduction

The number of adults with type 2 diabetes mellitus (T2DM) is increasing worldwide and if the trend continues the number will rise to 693 million by 2045^1^. An increase in the aging and obese population is one of the key factors associated with this rise and mitochondria have been identified as the locus of convergence for the multiple dysregulated pathways associated with aging, obesity and T2DM^2,3^.

The mitochondrial genome is a non-chromosomal DNA which plays an important role in a variety of homeostatic and signalling processes^4,5^. Unlike the nuclear genome, which normally has only two copies per cell, the mitochondrial genome contains multiple copies per cell^6^. Because of its close proximity with higher levels of reactive oxidative species (ROS), mitochondrial DNA (mtDNA) is prone to oxidative stress; which may lead to mitochondrial dysfunction, characterized by lowered oxidative capacity and reduction in energy production^7^. Mitochondrial dysfunction is associated with aging process and can affect cellular functions and thereby results in a variety of human diseases^8^ such as cancer^9,10^, neurodegenerative diseases^11,12^, cardiovascular diseases^10,13^, diabetes and metabolic syndrome^14,15^.

On the other hand, improvement in mitochondrial function, even after critical illness, has been shown to be associated with better survival^16^. Mitochondrial copy number (mtDNA-CN) is a surrogate marker of mitochondrial function^17^. Higher mtDNA-CN is a biomarker of better mitochondrial function and vice versa. Variations in mtDNA-CN are associated with the risk of T2DM; however, most studies used a case–control design and had published controversial results^18–25^. One of the reasons for conflicting results is suggested to be the methodology, as various analytical factors can affect the quantification of mtDNA copy number^17^. We have recently developed a well-optimized droplet digital PCR (ddPCR) based method for accurate quantification of mtDNA copy number^8^. Moreover, to the best of our knowledge, no study has so far investigated the relationship between mtDNA copy number and future risk of T2DM. In this study, we aimed to investigate the association of mtDNA-CN with both prevalent and incident T2DM events in a large population-based prospective study conducted on women.

## Materials and methods

### Study population

Data used in the present study were derived from the Women’s Health in Lund Area (WHILA), a population-based follow-up study. All women aged 50–59 years during 1995–2000 (born between 1935 and 1945) and that were living in any of the five southern municipalities in Sweden were invited to participate in a health survey. The regional ethical committee at Lund University approved the study (approval nos. 95/174, 2011/494 and 2015/6) and written informed consent was given by all the participants in the study after full explanation of the purpose and all the protocols were conducted in accordance with the Helsinki Declaration. From Dec 1995 to Feb 2000, a total of 6917 women (out of 10,766, the total population of women in in the five southern municipalities in 1995) underwent a physical examination and answered a questionnaire. The questionnaire that was distributed to all participants has been described previously^26^. To summarize, after written consent, the participants were given up to two hours to answer the questionnaire. If they had any uncertainties, they could ask an experienced research nurse. There was no financial reimbursement for participation. Participants were followed from the day of screening until death, or if no such event occurred until May 31st, 2015.

All T2DM diagnoses (prevalent and during follow-up) were obtained through (1) questionnaire, a composite of pre-existing and validated questionnaires^27^, (2) oral glucose tolerance test (OGTT), women with features of the metabolic syndrome (as defined previously)^28^ underwent a baseline OGTT 1–4 weeks after first screening and (3) clinical registers which includes, the Primary Health Care Register in Region Skåne; PASIS, in- and outpatient register, drug prescription register (ATC-code = A10) and death register.

Age at screening and BMI were used as continuous variables. Educational level was categorized into low ≤ 9 years, middle 10–12 and high > 12 years of schooling. Physical activity at home was defined according to the questionnaire and the participants with a score between 1 and 3 were categorized as low activity at home. 1 = hardly do anything at all. 2 = mostly sedentary. 3 = light physical exertion. High activity at home was categorized with a score between 4 and 6. 4 = strenuous exercise 1–2 h/week. 5 = strenuous exercise at least 3 h/week. 6 = hard regular exercise.

Alcohol consumption was assessed by the following question: ‘How much alcohol do you drink in an ordinary week?’ and to report the quantity of glass/bottles (specified in centilitres) of beer, wine and spirits respectively, or the option ‘no alcohol’. Each participant’s consumption of alcohol was converted into grams of alcohol for beer, wine and spirits separately and then summarized into total grams of alcohol per day. The women were divided in three groups: (1) 0 g, Women who did not drink any alcohol in an ordinary week.

(2) 0.1–11.9 g alcohol per day and (3) ≥ 12 g alcohol per day, as described previously^29^.

### Quantification of mtDNA copy number by ddPCR

Absolute quantification of mtDNA-CN was performed by our well optimized ddPCR based method, as described previously^8^. Briefly, total genomic DNA was extracted from whole blood (200 μL) using QIAamp 96 DNA Blood (Qiagen, Inc., Hilden, Germany) according to manufacturer’s instructions. Extracted DNA was frozen at − 20 °C for future use. For mtDNA quantification, primers and probes targeting the mitochondrially encoded NADH dehydrogenase 1 (*MT-ND1)* and for reference nuclear DNA (nDNA) quantification, primers and probes targeting the eukaryotic translation initiation factor 2C, 1 (*EIF2C1)* also known as argonaute 1, RISC catalytic component (Gene ID: 26523) were used. All primer and probes were obtained from Bio-Rad (Hercules, California, USA). Sequence and other information about primers and probes is available at www.bio-rad.com with the following ID numbers: MT-ND1 (assay ID: dHsaCPE5029120, sequence accession number: NC_012920.1 and EIF2C1 (assay ID: dHsaCP1000002, sequence accession number: NM_012199.2.

Probes targeting mtDNA were attached with FAM fluorophore whereas nuclear DNA targeting probes were attached with HEX and had Iowa Black FQ quencher on all probes. The ddPCR method was performed according to manufacturer’s instructions with some modifications as described below. First amplification was performed in a 20 µl multiplex reaction containing 1 ng of purified DNA from whole blood, 900 nM of primers and 250 nM of probes, 2X ddPCR supermix for probes (no UTP) and 5U/reaction HindIII enzyme (Thermo Scientific, Hudson, NH, USA) and was incubated for 20 min at room temperature to allow digestion with restriction enzyme (HindIII). Samples were subjected to droplet generation by an automated droplet generator and later end-point PCR was performed as described previously^8^. The PCR plate was incubated overnight at 4 °C. This additional step significantly improved the droplet recovery to maximum (19,000–20,000 droplets). Finally, droplets were read on droplet reader and data were analysed using QuantaSoft Software which determines the numbers of droplets that were positive and negative for each fluorophore in each sample. The fraction of positive droplets was then fitted to a Poisson distribution in QuantaSoft Software to determine the absolute copy number in units of copies/µl. DNA preparation and PCR experiments were performed in separate designated rooms and each run included negative and positive controls. No significant hazards or risks are associated with the reported work.

### Statistical analysis

MtDNA and other variables were measured at baseline (1995–2000) and T2DM was defined as either prevalent (diagnose date before/at baseline) or as incident (diagnose date after baseline).

We presented characteristic variables for all individuals and for mtDNA-CN categorized into quartiles (equal frequency grouping intervals to achieve categories with equal number of individuals), using mean and SD, median and IQR, and numbers and percentages. We tested the association between mtDNA-CN (categorized into quartiles) and each characteristic variable using a test for trend, i.e., ordinal logistic regression.

Further on, we presented the characteristic variables separately in two groups, prevalent and no prevalent T2DM, and tested the difference in characteristics between these groups using Student’s t-test, Chi-square test and Wilcoxon rank-sum test. Similarly, we presented and tested the characteristics at baseline between incident and no incident T2DM (prevalent diabetes excluded).

To test the association between mtDNA-CN at baseline and prevalent T2DM, we used linear regression analysis with mtDNA-CN (continuous) as the outcome. When testing the association between mtDNA-CN at baseline and incident T2DM, we instead used Cox-regression analysis with time to T2DM as outcome and mtDNA-CN (reversed and standardized) as exposure. We also tested different categorizations of mtDNA-CN as exposure (dichotomized and categorized into tertiles and into quartiles). Kaplan–Meier survival curves were calculated to estimate the probability of remaining free of T2DM in the two groups. Participants who were lost during follow-up were treated as censored observations. The difference between the survival curves was tested with the log-rank test. In a sensitivity analysis we excluded pre-diabetic individuals defined from the second screening based on impaired fasting glucose (IFG) and impaired glucose tolerance (IGT). Statistical analyses were performed by using STATA version 15 (StataCorp LP).

### Ethics approval and consent to participate

The regional ethical committee at Lund University approved the study (approval nos. 95/174, 2011/494 and 2015/6) and written informed consent was given by all the participants in the study after full explanation of the purpose and nature of all procedures.

## Results

**Figure 1:**
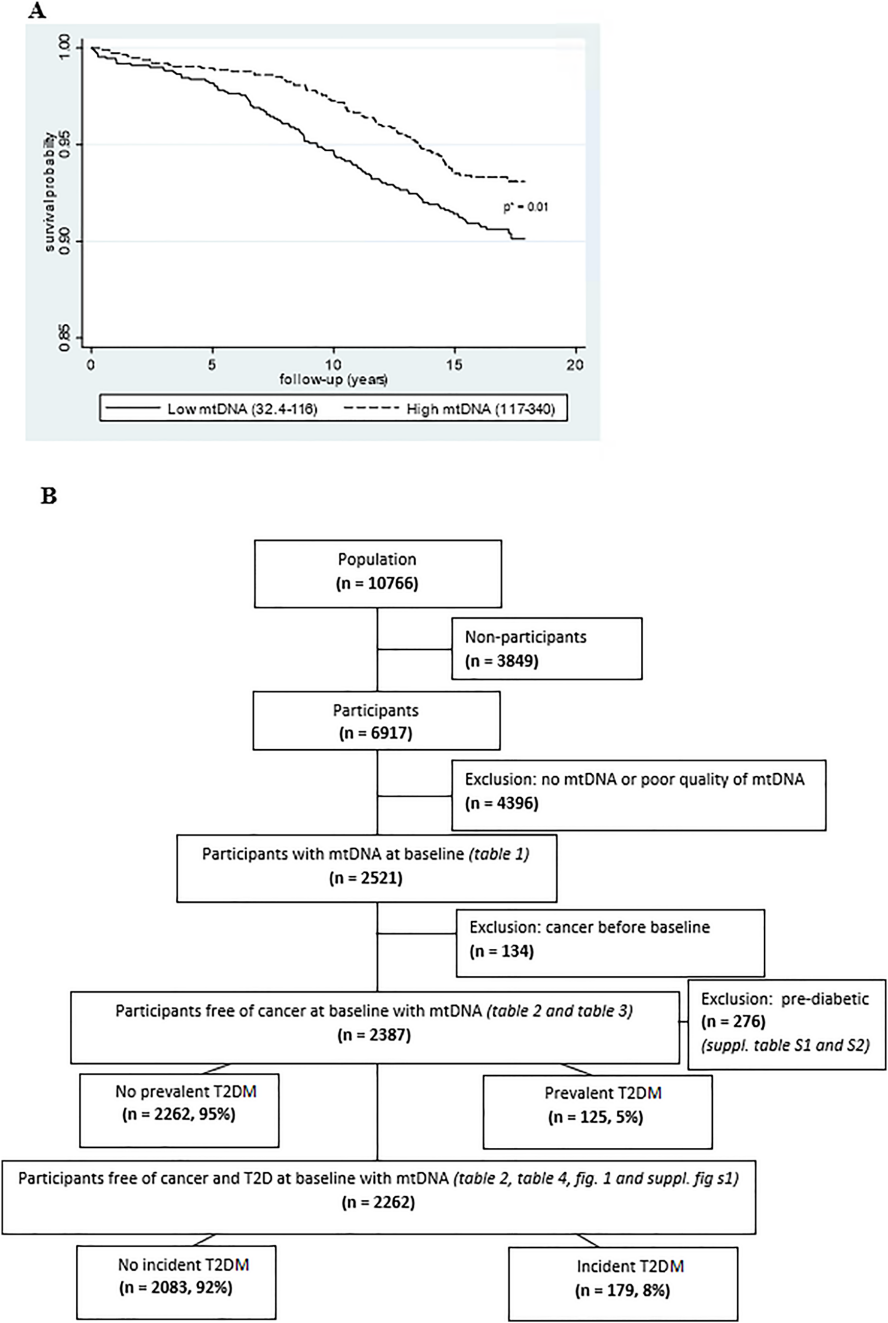
(**A**) Kaplan–Meier curves were plotted to calculate the probability of population free of T2DM during follow-up. Mitochondrial DNA-CN was dichotomized into high (dotted line) and low (continuous line) according to the median. *p value for difference in time to T2DM between low and high mtDNA (log-rank test). (**B**) Flowchart for population included in the study.

The study included middle-aged women population followed for 20 years (median follow-up 17 years). Blood samples for DNA analyses were collected only midway (October 1997) through the study and therefore were available for 3062 participants, out of which 541 samples were of poor quality of DNA as observed during ddPCR analysis of reference gene and where 134 participants had prevalent cancer, which were then excluded. Among the remaining 2387 participants, 125 (5%) participants had prevalent T2DM and among the 2262 participants without prevalent T2DM, 179 (8%) had incident T2DM (Fig. 1B, population flow chart).

### Characteristics of the whole population according to the mtDNA copy number

The mean age of the study population was 57 years at baseline. Comparison of variables according to the quartiles of mtDNA-CN and tested for trend using ordinal logistic regression showed the following; higher the mtDNA-CN the younger age (*p* = 0.01), the lower number of smokers (*p* < 0.0001), the higher number of heavy alcohol consumption (*p* = 0.01), the higher education (*p* = 0.002), the lower systolic blood pressure (*p* = 0.03)), the lower triglyceride levels (*p* = 0.001), the higher LDL-C (*p* = 0.04) and the HDL-C (*p* < 0.0001) and the better 2-h glucose tolerance (*p* = 0.01). BMI, physical activity, diastolic blood pressure and fasting glucose levels were not significantly associated with mtDNA-CN (*p* > 0.05) in the whole population (Table 1).

**Table 1:** Baseline characteristics of study subjects according to the levels of mtDNA-CN.

	Total (*n* = 2521)	mtDNA-CN categorized into quartiles				*p* values^a^
		Low, ≤ 99 (*n* = 618)	Medium low, 100–116 (*n* = 629)	Medium high 117–134 (*n* = 626)	High ≥ 135 (*n* = 648)	
MtDNA-CN, mean (SD)	118.1 (27.6)	84.8 (12.0)	108.1 (4.8)	125.0 (5.2)	152.8 (19.3)	–
Age, mean (SD)	57.0 (2.8)	57.1 (2.8)	57.1 (2.8)	56.9 (2.8)	56.8 (2.8)	0.01
BMI, mean (SD)	25.6 (4.1)	25.7 (4.2)	25.7 (4.2)	25.7 (4.1)	25.3 (3.9)	0.10
**Smoker**						
Yes or former/no	20/78	26/71	22/77	19/81	13/85	
%	19–22	23–23	19–26	16–22	10–16	
95% CI	78–81	69–77	74–81	78–84	84–90	< 0.0001
Alcohol consumption (grams per day), 0.0.1–11.9	24/57/13	26/56/12	26/57/12	24/58/12	22/58/15	
≥ 12	24–28	24–32	2–31	22–29	19–26	
%	59–63	55–63	56–64	58–66	57–65	
95%CI	12–15	10–16	10–16	10–16	13–19	0.01
Education, ≤ 9 years, 10–12	54/15/29	56/15/27	58/14/26	54/15/31	48/17/33	
> 12	53–57	53–61	55–62	50–58	45–53	
%	14–17	13–18	12–18	12–18	14–20	
95% CI	28–32	24–31	23–30	27–35	30–37	0.002
Activity at home, low/high	56/42	58/40	57/41	56/42	53/45	0.06
%	56–59	55–63	54–62	53–61	50–58	
95% CI	41–45	37–45	38–46	39–47	42–50	
Systolic blood pressure, (mmHg), mean (SD)	132.1 (17.2)	133.2 (18.2)	132.1 (17.3)	132.4 (16.9)	130.9 (16.2)	0.03
Diastolic blood pressure (mmHg), mean (SD)	85.1 (9.0)	85.0 (9.1)	85.3 (9.0)	85.1 (9.2)	84.7 (8.6)	0.48
Triglycerides (mmol/L), median (IQR)	1.51 (1.0)	1.59 (1.0)	1.54 (0.98)	1.5 (0.95)	1.42 (0.9)	0.001
HDL-C (mmol/L), mean (SD)	1.81 (0.45)	1.76 (0.45)	1.81 (0.45)	1.81 (0.45)	1.87 (0.44)	< 0.0001
LDL-C (mmol/L), mean (SD)	3.42 (0.98)	3.35 (1.0)	3.42 (0.94)	3.44 (0.97)	3.47 (1.0)	0.04
Fasting glucose (mmol/L), mean (SD)	5.0 (0.6)	5.0 (0.6)	5.1 (0.6)	5.0 (0.5)	5.1 (0.5)	0.72
2 h-glucose (mmol/L), mean (SD)	6.1 (1.7)	6.2 (1.7)	6.2 (2.0)	6.0 (1.7)	5.9 (1.4)	0.01

^a^Test for association between mtDNA-CN and characteristic using ordinal logistic regression.

### Prevalent T2DM

Participants with cancer diagnosis were excluded from further analyses (see flowchart in Fig. 1B). Among the remaining 2387 participants, a total of 125 (5%) had prevalent T2DM. Compared to participants without prevalent T2DM, participants with prevalent T2DM were older (mean ± SD age = 56.9 ± 2.8 vs. 58 ± 2.8, *p* < 0.0001), had higher BMI (mean ± SD = 25.5 ± 3.9 vs. 28.4 ± 5.4, *p* < 0.0001), had lower education (30% vs. 19%, > 12 years education, *p* = 0.04) and physical activity (43% vs. 30% of higher activity, *p* = 0.005), higher systolic (mean ± SD = 131.6 ± 17 vs. 140 ± 19.1 mmHg, *p* < 0.0001) and diastolic blood pressures (mean ± SD = 85.0 ± 8.9 vs. 87.3 ± 9.5 mmHg, *p* = 0.005), higher triglycerides [median (IQR) = 1.51 (0.97) vs. 1.91 (1.32) mmol/L, *p* < 0.0001] and LDL-C (mean ± SD = 3.41 ± 0.98 vs. 3.61 ± 0.98, *p* = 0.04) levels, lower HDL-C (mean ± SD = 1.82 ± 0.45 vs. 1.59 ± 0.46 mmol/L, *p* < 0.0001) levels and higher fasting glucose (mean ± SD = 5.0 ± 0.5 vs. 5.9 ± 0.9 mmol/L, *p* < 0.0001) levels and poor 2 h glucose tolerance (mean ± SD = 5.9 ± 1.4 vs. 8.6 ± 3 mmol/L, *p* < 0.0001), (Table 2). Smoking and alcohol consumption were not associated with prevalent T2DM (*p* ≥ 0.05, Table 2). Mitochondrial DNA-CN was significantly lower in prevalent T2DM (mean ± SD; 111 ± 24 copies/µL) compared to participants without prevalent T2DM (mean ± SD; 118 ± 27 copies/μL), *p* = 0.003 (Table 2). We also categorized mitochondrial levels into low, medium low, medium high and high categories and found that 26% of participants with no prevalent T2DM comprised the high category compared to 18% participants with prevalent T2DM, *p* = 0.05 (data not shown).

**Table 2:** Baseline characteristics and mtDNA-CN levels in study subjects with and without prevalent and incident T2DM.

	No prevalent T2D (*n* = 2262, 95%)	Prevalent T2D (*n* = 125, 5%)	*p* values^a^	No incident T2D (*n* = 2083, 92%)	Incident T2D (*n* = 179, 8%)	*p* values^b^
MtDNA-CN, mean (SD)	118 (27)	111 (24)	0.003	119 (27)	113 (23)	0.006
Age, mean (SD)	56.9 (2.8)	58.0 (2.8)	< 0.0001	56.9 (2.8)	57.2 (3.0)	0.17
BMI, mean (SD)	25.5 (3.9)	28.4 (5.4)	< 0.0001	25.2 (3.8)	28.5 (4.6)	< 0.0001
Smoker, yes or former/no	20/78	18/79	0.71	20/78	21/79	0.85
%	18–22	12–26		18–22	15–28	
95% CI	76–80	71–86		76–80	72–85	
Alcohol consumption (grams per day), 0.0.1–11.9	25/58/13	30/54/10	0.20	23/58/13	27/57/12	0.56
≥ 12	23–27	22–39		21–25	20–34	
%	56–60	45–63		56–60	49–64	
95% CI	12–14	6–17		12–15	7–17	
Education, ≤ 9 years, 10–12	54/15/30	60/18/19	0.04	53/15/30	63/14/22	0.03
> 12	52–56	51–69		51–55	56–70	
%	14–17	12–26		13–17	9–20	
95% CI	28–32	13–27		28–32	16–29	
Activity at home, low/high	55/43	68/30	0.005	55/43	59/39	0.26
%	53–57	59–76		53–57	52–66	
95% CI	41–45	22–39		41–45	32–47	
Systolic blood pressure, (mmHg), mean (SD)	131.6 (17.0)	140.0 (19.1)	< 0.0001	130.8 (16.7)	140.7 (17.1)	< 0.0001
Diastolic blood pressure (mmHg), mean (SD)	85.0 (8.9)	87.3 (9.5)	0.005	84.6 (8.9)	89.1 (8.0)	< 0.0001
Triglycerides (mmol/L), median (IQR)	1.51 (0.97)	1.91 (1.32)	< 0.0001	1.47 (0.93)	1.86 (1.05)	< 0.0001
HDL-C (mmol/L), mean (SD)	1.82 (0.45)	1.59 (0.46)	< 0.0001	1.84 (0.44)	1.60 (0.44)	< 0.0001
LDL-C (mmol/L), mean (SD)	3.41 (0.98)	3.61 (0.98)	0.04	3.39 (0.98)	3.53 (0.97)	0.10
Fasting glucose (mmol/L), mean (SD)	5.0 (0.5)	5.9 (0.9)	< 0.0001	4.9 (0.4)	5.3 (0.5)	< 0.0001
2 h-glucose (mmol/L), mean (SD)	5.9 (1.4)	8.6 (3.0)	< 0.0001	5.7 (1.3)	7.0 (1.6)	< 0.0001

^a^Test for difference between no prevalent and prevalent T2DM using Student’s t-test, Chi-square test and Wilcoxon rank-sum test.

^b^Test for difference between no incident and incident T2DM using Student’s t-test, Chi-square test and Wilcoxon rank-sum test.

To investigate the association between prevalent T2DM and mtDNA-CN, we performed univariate and adjusted linear regression analysis with mtDNA-CN as an outcome. Our results show that prevalent T2DM was associated with lower mtDNA-CN (β =  −7.36, 95% CI − 12.2; − 2.5), *p* = 0.003. However, this association decreased after adjusting for age, BMI, education, smoking status and physical activity (β =  −4.86, 95% CI − 9.8; 0.11), *p* = 0.06 (Table 3). In a sensitivity analysis where pre-diabetic individuals (IFG and IGT) were excluded, the association between T2DM and lower mtDNA remained significant in univariate (β =  −10.8, 95% CI − 16.2; − 5.5, *p* < 0.0001 as well as in multivariable analysis (β =  −8.46, 95% CI − 14.0; − 2.92, *p* = 0.003 (Table 5). Baseline characteristics of the study population included in sensitivity analysis are shown in (Supplementary Table 1).

**Table 3:** Association between prevalent T2DM and mtDNA-CN using linear regression models.

Dependent variable: mtDNA-CN	Univariate		Adjusted^a^	
	*β* (*p* value)	95% CI	*β* (*p* value)	95% CI
Prevalent T2DM (yes vs. no)	− 7.36 (0.003)	− 12.2; − 2.5	− 4.81 (0.06)	− 9.8; 0.17
Age	− 0.51 (0.009)	− 0.90; − 0.13	− 0.43 (0.03)	− 0.83; − 0.04
BMI	− 0.30 (0.03)	− 0.56; − 0.03	− 0.26 (0.06)	− 0.54; 0.01
Smoker (yes or former vs. no)	− 7.62 (< 0.0001)	− 10.3; − 4.92	− 7.63 (< 0.0001)	− 10.4; − 4.90
Education (≤ 9 years vs. ≥ 10 years)	− 3.86 (0.001)	− 6.0; − 1.7	− 2.68 (0.02)	− 4.9; − 0.46
Activity at home (low vs. high)	− 1.87 (0.10)	− 4.09; 0.34	1.10 (0.34)	− 1.1; 3.3

^a^Adjusted for all variables (prevalent T2DM, age, BMI, smoking, education and activity at home).

Cancer at or before baseline were excluded.

### Incident T2DM

Participants free of prevalent T2DM and cancer (n = 2262) were followed up for a median of 17 years, with 179 incident T2DM events. Baseline characteristics (at the time of inclusion in the study, 1995–2000) of participants with incident T2DM and no T2DM during follow-up are shown in Table 2.

Level of mtDNA-CN were significantly higher in those with non-incident T2DM (copies/μL, mean ± SD; 119 ± 27) compared to those with incident T2DM (113 ± 23), *p* = 0.006. Participants diagnosed with T2DM during follow-up had significantly higher BMI, systolic and diastolic blood pressures, triglycerides, lower education, fasting glucose and poor glucose tolerance compared to participants free of T2DM during follow-up (*p* < 0.05, Table 2). In contrast, HDL-C and mtDNA-CN were significantly lower in participants diagnosed with T2DM during follow-up (*p* < 0.05, Table 2). Since participants diagnosed with incident T2DM had lower education and it was previously linked to higher BMI and lower physical activity, we investigated it in our study. Our results showed that lower education was associated with higher risk of incident T2DM (OR = 1.50, *p* = 0.01) independent of physical activity (OR = 1.50, *p* = 0.02) but was partly explained by BMI (OR = 1.33, *p* = 0.09) (data not shown). Incident T2DM was associated with levels of mtDNA. For one standard deviation decrease in mtDNA, the risk (or hazard) for incident T2DM increased 1.25 times (Hazard ratio, HR = 1.25, 95% CI 1.07–1.45). This association remained significant even after adjusting for age, BMI, smoking status, education and physical activity (HR = 1.20; 95% CI 1.02–1.40), Table 4. The mtDNA-CN was also categorized according to median, tertiles and quartiles. Compared to highest quartile (reference), the lowest quartile was associated with significantly higher risk of incident T2DM (HR = 1.78, 95% CI 1.11–2.83, *p* = 0.02) independent of age, BMI, smoking status, education and physical activity (Table 4). Results on median and tertiles of mtDNA-CN showed similar results and are presented in Table 4. Kaplan–Meier survival curves were plotted to estimate the probability of participants remaining free of T2DM during follow-up by dividing mtDNA-CN according to median into low (≤ 116) and high (> 116) copies/µL. Participants with higher mtDNA-CN had lower probability of having T2DM during follow-up compared to those who had lower mtDNA-CN (log-rank test, *p* = 0.01), Fig. 1A (Table 5).

**Table 4:** Hazard ratios for incident T2DM per 1 standard deviation decrease in mtDNA-CN and according to median, tertiles and quartiles of mtDNA-CN in study subjects.

Dependent variable: Time to incident T2DM	Univariate		Adjusted^a^	
	HR^b^ (*p* value)	95% CI	HR^b^ (*p* value)	95% CI
MtDNA-CN (decrease, standardised)^c^	1.25 (0.004)	1.07; 1.45	1.20 (0.02)	1.02; 1.40
MtDNA-CN median^d^ (low vs. high)	1.45 (0.015)	1.07; 1.94	1.43 (0.02)	1.05; 1.95
**MtDNA-CN tertile**^**e**^				
Low	1.54 (0.02)	1.06; 2.23	1.50 (0.04)	1.02; 2.22
Medium	1.50 (0.03)	1.03; 2.18	1.48 (0.048)	1.00; 2.19
High (ref)	1		1	
**MtDNA-CN quartile**^**f**^				
Low	1.88 (0.006)	1.20; 2.93	1.78 (0.02)	1.11; 2.83
Med low	1.79 (0.01)	1.15; 2.80	1.75 (0.02)	1.10; 2.79
Med high	1.56 (0.06)	0.98; 2.46	1.45 (0.13)	0.90; 2.35
High (ref)	1		1	

^a^Adjusted for age, BMI, smoking, education and physical activity.

^b^Hazard ratio for risk of incident T2DM.

^c^MtDNA has been reversed and standardized (HR for a one standard deviation decrease in mtDNA).

^d^Low mtDNA = 32.4–116, high mtDNA = 117–340.

^e^Low mtDNA = 32.4–105, medium mtDNA = 106–127, high mtDNA = 128–340.

^f^Low mtDNA = 32.4–99, medium low mtDNA = 100–116, medium high mtDNA = 117–134, high mtDNA = 135–340.

Prevalent cancer and prevalent T2DM were excluded. MtDNA-CN, Mitochondrial DNA copy number.

T2DM = type 2 diabetes mellitus; HR = hazard ratio; CI = confidence interval.

**Table 5:** Sensitivity analysis.

Dependent variable: mtDNA-CN	Univariate		Adjusted^a^	
	β (*p* value)	95% CI	β (*p* value)	95% CI
Prevalent T2DM (yes vs. no)	− 10.8 (< 0.0001)	− 16.2; − 5.5	− 8.46 (0.003)	− 14.0; − 2.92
Age	− 0.51 (0.016)	− 0.93; − 0.09	− 0.37 (0.09)	− 0.80; 0.06
BMI	− 0.24 (0.11)	− 0.54; 0.06	− 0.17 (0.30)	− 0.48; 0.15
Smoker (yes or former vs. no)	− 7.19 (< 0.0001)	− 10.1; − 4.30	− 7.08 (< 0.0001)	− 10.0; − 4.16
Education (≤ 9 years vs. ≥ 10 years)	− 4.37 (< 0.0001)	− 6.7; − 2.0	− 3.17 (0.009)	− 5.6; − 0.78
Activity at home (low vs. high)	− 2.19 (0.07)	− 4.55; 0.18	1.36 (0.27)	− 1.0; 3.8

Linear regression models examining effect of prevalent T2DM on mtDNA-CN after excluding pre-diabetic subjects based on IFG and IGT.

^a^Adjusted for all variables (prevalent T2DM, age, bmi, smoking, education and physical activity).

Cancer at or before baseline and pre-diabetics (n = 276) subjects based on IFG (impaired fasting glucose) and IGT (impaired glucose tolerance were excluded).

We further explored to identify which variables that affected the association between mtDNA-CN and T2DM the most. We did this by adding the variables one by one in the univariate model with time to incident T2DM as outcome and continuous mtDNA-CN as exposure. Our results showed that adding smoking in the model decreased the association between mtDNA-CN and T2DM by 11%. Other variables which decreased the association between mtDNA-CN and T2DM were the following: BMI by 9.8%, education by 9% age by 7%, and physical activity by 3%. Further analysis on incident T2DM showed that smoking had a modifying effect on the association between mtDNA-CN and incident T2DM (interaction effect: HR = 2.5, 95% CI = 1.03; 6.1), i.e., the effect of low mtDNA-CN on the risk of incident T2DM was significantly higher for smokers compared to non-smokers. Kaplan–Meier curve analysis with stratification by mtDNA-CN and smoking status also demonstrated this modifying effect. Women who were smokers and had low baseline mtDNA-CN had higher probability of having T2DM (lower survival probability) during follow-up compared to smokers with high baseline mtDNA-CN (*p* value from a log-rank test = 0.01), (Supplementary Figure S1).

## Discussion

We investigated the potential role of mtDNA-CN number in T2DM in 2387 participants from a population-based study conducted on middle-aged women. The key findings from this study were that the low mtDNA-CN was associated with both prevalent and incident T2DM. The association between mtDNA-CN remained significant for incident T2DM even after adjusting for the covariates. The association between mtDNA-CN and prevalent T2DM decreased after adjusting for the covariates, but a sensitivity analysis showed that, after excluding the pre-diabetic cases, the association became stronger, indicating that pre-diabetic cases may distort the “true” relationship between mtDNA-CN and T2DM. To the best of our knowledge, this is the first population-based follow-up study in which the role of mtDNA copy number as a possible predictor of incident T2DM has been investigated.

Even though several studies have investigated a potential role of mtDNA-CN in prevalent T2DM^19,22–25^, the results are conflicting. Methodology is one of the major factors attributed to these conflicting results^17^. We have recently developed a well-optimized ddPCR method by taking into account several important analytical factors which may affect the accurate quantification of mtDNA copy number^8^. Our results on prevalent T2DM are in-line with the majority of previously published results^24,25,30^. However, key question remains whether the decrease in mtDNA copy number is a cause or consequence of T2DM. Our results demonstrate that participants with low mtDNA-CN at baseline are associated with a higher risk of future T2DM events. Furthermore, this association remained significant even after adjusting for other risk factors for T2DM such as age, BMI, physical activity, education and smoking status.

We also found significant associations between mtDNA-CN and smoking and lipid profile parameters. Cigarette smoking is one of the most important modifiable risk factors for T2DM^31^ and may accelerate micro and macrovascular complications associated with the T2DM^32,33^. Even though the regular exposure to smoking is associated with the increased risk of T2DM, the prevalence of smoking among people with T2DM appears to be similar to that of the general population^34^, which is also observed in this study. Interestingly, we found a strong association between smoking and mtDNA-CN as well as between smoking and T2DM. The association between mtDNA-CN and T2DM was confounded by other factors known to be associated with T2DM and smoking showed the maximum confounding effect. To explore whether smoking has an effect on the association between mtDNA-CN and T2DM, we performed an analysis with an interaction term between mtDNA-CN and smoking status and found that smoking had a modifying effect on the association between mtDNA-CN and incident T2DM. Women who were smokers had a significantly higher effect of low mtDNA-CN on risk for T2DM compared to women who were non-smokers. This interesting finding needs further investigation and may partly explain the cause-effect link between smoking and T2DM which is not yet well established. Nevertheless, given the evidence linking mitochondrial dysfunction with aging, insulin resistance and T2DM, it is important to emphasize that improving mitochondrial bio-energetic functions may reduce the incidence of T2DM and co-morbidities associated with it. For example, restoring mitochondrial bio-energetic functions have been associated with up-regulation of genes involved in mitochondrial biogenesis and survival^35^. Therefore, improving life-style such as quitting smoking^36^, starting to exercise^37^ and having a healthy diet^16^ may possibly improve mitochondrial function and thereby decrease the risk of many diseases.

Lower levels of education were significantly associated with both lower mtDNA-CN and higher risk of T2DM. In agreement with our results, lower education has been linked with higher risk of T2DM that is partly explained by higher BMI and less physical activity^38^. In our study, the association between T2DM and education was independent of physical activity, but not of BMI. Mitochondrial dysfunction is also associated with obesity^39^ and lower physical activity^40^. Interestingly, we found that mtDNA-CN was associated with education independent of both BMI and physical activity. Therefore the association between education and mtDNA-CN in our study is not explained by BMI or physical activity.

Mitochondria are a key regulator in energy homeostasis as it is the primary site of adenosine triphosphate production. Mitochondrial dysfunction, a hallmark of the aging process, may play a critical role in insulin resistance, a primary component in T2DM pathophysiology^30^. The mtDNA-CN is a well-known surrogate biomarker of mitochondrial function. We found an inverse association between glucose intolerance and mtDNA-CN, thus further supporting an important role for mitochondrial dysfunction in T2DM pathophysiology. One possible mechanism behind this association could be mitochondrial dysfunction in pancreatic beta-cells which may negatively affect formation of coupling factors, dynamics of cellular Ca^2+^, and rise in the ATP/ADP^41^. Taken together these may eventually lead to insulin resistance, however, this needs to be confirmed in future studies.

Although this study was not designed to address the mechanism behind the association between mtDNA-CN and T2DM, a possible mechanism for a decrease in mtDNA-CN in diseases can be due to accumulations of mutations in the mitochondrial genome which may lead to mitochondrial dysfunction^17^. Secondly, it could be due to mtDNA’s close proximity with a high concentration of reactive oxidative species (ROS) produced in the mitochondrial matrix, which may lead to mitochondrial dysfunction^7^. These factors may together significantly affect the function of mitochondria and thereby result in lower number of mtDNA-CN in some diseases. Mutations in the mitochondrial genome and their associations with mitochondrial dysfunction in T2DM therefore warrant further investigation.

### Strengths and weaknesses of the study

This study has several strengths and limitations that must be recognised in the interpretation of our results. Firstly, this study includes a large sample size recruited from a population-based study with a long follow-up, secondly the homogenous study sample in terms of age and sex, thirdly it includes both prevalent and incident T2DM and we have quantified the absolute copy number of mtDNA by a well-optimized ddPCR method considering several technical factors which may affect mtDNA quantification. Cases of T2DM were defined from questionnaire, prescription register, second screening (OGTT), in- and outpatient and death register, this means that we included all women diagnosed with T2DM in our study. We identified 5% prevalent and 8% incident T2DM which is in accordance with the prevalence and projected future T2DM in women in Sweden respectively^42^. Our study also has several limitations such as our sample was based only on women participants; the generalizability of the current findings still needs to be established by testing among men. Finally, we only had DNA samples from half of the study population, however, due to the population-based design of the study, it is expected that participants included in this study represent the whole population.

## Conclusions

Both prevalent and incident T2DM were associated with low mtDNA-CN in a large population-based follow-up study on women. If confirmed in other settings, low mtDNA copy number can be a predictor of the risk of T2DM.

## Supplementary Information

Supplementary Table S1.

Supplementary Figure Legend.

Supplementary Figure S1.

## Data Availability

The data that support the findings of this study are available from the Swedish National Board of Health and Welfare; however, restrictions apply to the availability of these data, which were used under license for the current study, and so they are not publicly available.
