# Supplementary material for: Association of mitochondrial DNA copy number with prevalent and incident type 2 diabetes in women: A population-based follow-up study
Source: Sci Rep. 2021 Feb 25;11:4608. doi: 10.1038/s41598-021-84132-w (PMC7907271; doi:10.1038/s41598-021-84132-w)
Supplement: Supplementary file 1 — Supplementary Table S1. [file 41598_2021_84132_MOESM1_ESM.docx]

**Association of mitochondrial DNA copy number with prevalent and incident type 2 diabetes in women: A population-based follow-up study**

*Ashfaque A Memon, Jan Sundquist, Anna Hedelius, Karolina Palmér, Xiao Wang, Kristina Sundquist*

**Sensitivity analysis**

**Pre-diabetics excluded, n = 276**

**Supplementary Table 1.** Characteristics at baseline stratified by prevalent and no prevalent T2DM

|  | *No prevalent T2DM*  *(n = 2007, 95 %)* | *Prevalent T2DM*  *(n = 104, 5 %)* | *p-values^a^* |
| --- | --- | --- | --- |
| Age, mean (SD) | 56.8 (2.8) | 58.1 (2.7) | < 0.0001 |
| BMI, mean (SD) | 25.1 (3.9) | 27.8 (5.4) | < 0.0001 |
| Smoker, %  Yes or former/no | 20/78 | 18/79 | 0.68 |
| Alcohol consumption  (grams per day), %  0,0.1-11.9, >= 12 | 23/59/13 | 31/55/10 | 0.18 |
| Education, %  <= 9 years, 10-12, > 12 | 53/15/31 | 63/15/19 | 0.04 |
| Activity at home, %  Low/high | 54/44 | 67/31 | 0.008 |
| Systolic blood pressure, (mmHg), mean (SD) | 130.5 (16.6) | 140.0 (19.0) | < 0.0001 |
| Diastolic blood pressure (mmHg), mean (SD) | 84.4 (8.7) | 86.7 (9.6) | 0.007 |
| Triglycerides (mmol/L), median (IQR) | 1.64 (0.85) | 2.11 (1.12) | < 0.0001 |
| HDL (mmol/L), mean (SD) | 1.85 (0.44) | 1.61 (0.46) | < 0.0001 |
| LDL (mmol/L), mean (SD) | 3.41 (0.98) | 3.64 (1.01) | 0.03 |
| Fasting glucose (mmol/L), mean (SD) | 4.9 (0.4) | 5.7 (0.9) | < 0.0001 |
| 2h-glucose (mmol/L), mean (SD) | 5.3 (0.9) | 8.5 (3.3) | < 0.0001 |
| MtDNA-CN, mean (sd) | 119 (27) | 108 (23) | < 0.0001 |
| MtDNA-CN median^b^, %  Low/high | 48/52 | 64/36 | 0.001 |
| MtDNA-CN tertile^c^, %  Low/medium/high | 32/32/36 | 48/30/22 | 0.001 |
| MtDNA-CN quartile^d^, %  Low/med low/med high/high | 24/24/25/27 | 35/30/21/14 | 0.005 |

^a^Test for difference between no prevalent and prevalent T2DM using Student’s t-test, Chi-square test and Wilcoxon rank-sum test.

^b^Low mtDNA = 32.4-116, high mtDNA = 117-340

^c^Low mtDNA = 32.4-105, medium mtDNA = 106-127, high mtDNA = 128-340

^d^Low mtDNA = 32.4-99, medium low mtDNA = 100-116, medium high mtDNA = 117-134, high mtDNA = 135-340

Cancer at or before baseline and pre-diabetics (n = 276) subjects based on IFG (impaired fasting glucose) and IGT (impaired glucose tolerance were excluded.
