# Supplementary material for: Association of mitochondrial DNA copy number with prevalent and incident type 2 diabetes in women: A population-based follow-up study
Source: Sci Rep. 2021 Feb 25;11:4608. doi: 10.1038/s41598-021-84132-w (PMC7907271; doi:10.1038/s41598-021-84132-w)
Supplement: Supplementary file 2 — Supplementary Figure Legend. [file 41598_2021_84132_MOESM2_ESM.docx]

**Supplementary Figure S1.** Kaplan-Meier curves were plotted to calculate the probability of having T2DM in smokers and non-smokers according to the levels of mtDNA-CN. Mitochondrial DNA-CN was stratified into quartiles and effect of smoking was investigated on the probability of having T2DM during follow-up in smokers and non-smokers.
