# Supplementary figures and images for: Association of mitochondrial DNA copy number with prevalent and incident type 2 diabetes in women: A population-based follow-up study
Source: Sci Rep. 2021 Feb 25;11:4608. doi: 10.1038/s41598-021-84132-w (PMC7907271; doi:10.1038/s41598-021-84132-w)

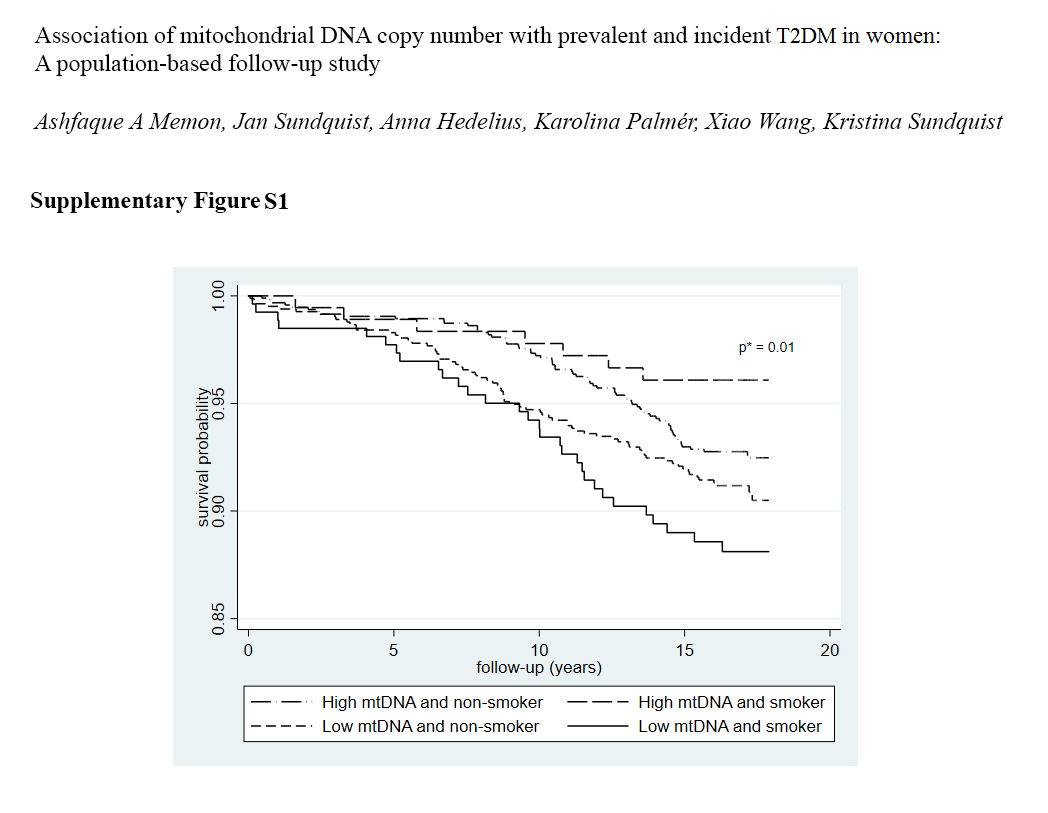

Supplement: Supplementary file 3 — Supplementary Figure S1. [file 41598_2021_84132_MOESM3_ESM.tif]
